# Proportion of upper extremity musculoskeletal disorders attributable to personal and occupational factors: results from the French Pays de la Loire study

**DOI:** 10.1186/s12889-020-08548-1

**Published:** 2020-04-06

**Authors:** Aboubakari Nambiema, Sandrine Bertrais, Julie Bodin, Natacha Fouquet, Agnès Aublet-Cuvelier, Bradley Evanoff, Alexis Descatha, Yves Roquelaure

**Affiliations:** 1 Univ Angers, CHU Angers, Univ Rennes, Inserm, EHESP, Irset (Institut de recherche en santé, environnement et travail) - UMR_S 1085, F-49000 Angers, France; 2 Santé publique France, the French national public health agency, Direction of Occupational Health, EpiprevTMS team associated to the University of Angers, F-49000 Angers, France; 3 INRS, Département Homme au travail, 1 rue du Morvan CS60027, 54519 Vandoeuvre, France; 4 Division of General Medical Sciences, Washington University School of Medicine, St. Louis, St. Louis, MO 13 63310 USA; 5 Inserm, UMS 011, unité cohortes épidémiologiques en population, Villejuif, France

**Keywords:** Risk factors, Population attributable fraction, Upper extremity musculoskeletal disorders

## Abstract

**Background:**

Upper extremity musculoskeletal disorders (UEMSD) are one of the most common and costly occupational health problems. We aimed to assess the population-attributable fraction (PAF) of personal and occupational risk factors associated with incident UEMSD in a working population.

**Methods:**

From 2002 to 2005, a random sample of 3710 workers from the Pays de la Loire region in France, aged 20–59 were included by occupational physicians (OPs). Between 2007 and 2010, 1611 workers were re-examined by their OPs. Subjects free from UEMSD at baseline were included in this study (1275 workers, mean age: 38.2 years). Cox regression models with equal follow-up time and robust variance estimates were used to estimate age-adjusted and multivariable-adjusted relative risks (RRs) and their 95% confidence intervals (CIs). Based on multivariable models, PAF associated with each factor included in the models was estimated.

**Results:**

During the follow-up period, 143 (11%) cases of UEMSD were diagnosed. PAFs for factors associated with the incident UEMSD risk were 30% (7 to 51) for high physical exertion (RPE Borg scale ≥12), 12% (− 0.2 to 24) for low social support, 7% (− 3 to 17) for working with arms above shoulder level (≥2 h/day), 20% (12 to 28) for age group ≥45, 13% (3 to 22) for the age group 35–44, and 12% (0.3 to 24) for female gender.

**Conclusions:**

Our study suggests that an important fraction of UEMSD can be attributed to occupational exposures after the contributions of personal and other work-related factors are considered. In terms of public health, our findings are in agreement with the ergonomic literature postulating that a high proportion of UEMSD are preventable through modifying workplace risk factors. Such information is useful to help public health practitioners and policy makers implement programs of prevention of UEMSD in the working population.

## Background

Work-related Upper Extremity Musculoskeletal Disorders (UEMSD), which include peripheral nerve entrapments and tendon disorders, as well as nonspecific musculoskeletal regional pain disorders, are the main source of morbidity and work disability in the working populations of industrialized and developing countries [1–3]. UEMSD are a major cause of occupational disease leading to considerable human and socio-professional cost in terms of pain and discomfort in work and daily life, sometimes irreversible functional sequelae, reduced ability for work, and the risk of work disability [3–5].

The World Health Organization (WHO) defined work-related diseases as multifactorial diseases in which the work environment and the performance of work contribute significantly to the causation of the disease [6]. There is a broad consensus on the multifactorial nature of UEMSD, where both non-occupational factors and occupational factors interact in etiology and prognosis [7–18]. Most personal susceptibility attributes (e.g. age) cannot be modified by prevention interventions or medical interventions, in contrast to potentially modifiable systemic conditions (e.g., obesity) [19, 20]. Exposure to work-related biomechanical factors (e.g., repetitive movements, forceful manual exertion) and psychosocial factors (e.g., psychological job demand, social support) could be modified by workplace-based interventions [20–23].

Identifying the modifiable risk factors for UEMSD in the workplace with the highest impact can help public health practitioners and policy makers to better target interventions at the working population level [24]. In this context, the effect of a particular risk factor depends not only on the strength of the association between the risk factor and the disease, but also on the prevalence of the risk factor. Nevertheless, when associations between a disease and a risk factor are assessed using classical statistical measures (relative risk or odds ratio), the population effect of some factors associated with high values of these estimates may be overestimated if few people are actually exposed to these factors [25, 26]. Confounding issues should also be considered in the assessment of a risk factor effect due to the multifactorial origin of disease.

The population attributable fraction (PAF) [27] is now a commonly used measure of the population-level contribution of a risk factor on a disease. This approach has the advantage of simultaneously considering the prevalence of the exposure to risk factors within the population and their associations with the disease. Moreover, the PAF can be computed using a multivariable approach to quantify the relative impact of one or more work-related exposures, or even co-exposure, on the occurrence of UEMSD [28–33]. Assuming that other risk factors remain unchanged and that there is a causal relationship between the risk factors and UEMSD, the partial PAF [34] describes the proportion of UEMSD that could be prevented if exposure to modifiable risk factor(s) is reduced from the target working population [26]. Such information may provide an estimation of the theoretical maximum potential impact of prevention programs in the workplace [35]. The partial PAF is appropriate when the disease of interest is multifactorial, and other risk factors are not expected to change as a result of the hypothetical intervention [36]. This contribution assessment method of some risk factors to the disease burden at the population level is widely used in studies of cancer [37–44], diabetes [25], cardiovascular disease [45, 46] and hypertension [47] studies.

To date, to the best of our knowledge, no prospective study has estimated the partial PAF of work-related exposures for UEMSD in a working population. Such information may be useful to estimate the proportion of theoretically preventable UEMSD and improve prevention of UEMSD in the working population. Therefore, the aim of this study was to determine the partial PAF related to personal and occupational factors for UEMSD using the French Cosali cohort.

## Methods

### Study population

The current study used data from the Cosali cohort, which focused on musculoskeletal disorders (MSD) and working conditions among workers in the Pays de la Loire region. Between 2002 and 2005, 3710 subjects (2161 men, 1549 women) with a mean age of 38.7 (standard deviation [SD] = 10.3) were included. Data on personal characteristics and working conditions were collected by a self-administered questionnaire. Participants underwent a clinical examination performed by occupational physicians (OPs) in charge of the medical surveillance of salaried workers. The OPs were trained to perform the standardized clinical examination according to the European consensus criteria for evaluating the work-relatedness of UEMSD [48]. Medical conditions, such as rheumatoid arthritis and diabetes mellitus, were collected during this clinical examination. During the follow-up period between 2007 and 2010, 1611 workers were re-examined by their OP using the same procedure as for inclusion. See [49] for more details.

Each worker provided informed written consent to participate in this study and the study received approval from France’s Advisory Committee on the Processing of Information in Health Research (“CCTIRS”) and the National Committee for Data Protection (“CNIL”), first in 2001 and again in 2006.

### Outcome definition

Using the European consensus criteria for evaluating the work-relatedness of UEMSD [48], incident cases of UEMSD were defined as workers free of the six following clinically diagnosed UEMSD at baseline, and having at least one of them diagnosed at the follow-up: 1-Rotator cuff syndrome (RCS), 2-Lateral epicondylar tendinopathy (LET), 3-Carpal tunnel syndrome (CTS), 4-Ulnar tunnel syndrome, 5-Flexor-extensor peritendinitis or tenosynovitis of the forearm-wrist region, and 6-De Quervain’s tenosynovitis. Details about these disorders have been previously described [50].

### Assessment of potential risk factors

Three groups of potential risk factors were assessed at baseline: personal, biomechanical and psychosocial factors.

1. ***Personal factors*** included gender, age divided into three categories (< 35, 35–44 and ≥ 45 years), overweight/obesity (body mass index (BMI) ≥25.0 kg/m^2^), using the World Health Organization criteria [51]), rheumatoid arthritis (yes/no) and diabetes mellitus status (yes/no).
2. ***Biomechanical factors*** (using the European consensus criteria [48]): high repetitiveness of tasks (≥4 h/day), use of vibrating hand tools (≥2 h/day), repeated/sustained elbow movements (flexion/extension) (≥2 h/day), repeated/sustained posture with arms above shoulder level (≥2 h/day), pronation and supination movements (≥2 h/day), wrist twisting movements (≥2 h/day), and use of the pinch grip (≥4 h/day). The definition of exposure to repeated/sustained posture with shoulder abduction included, as previously defined [49], workers who reported being exposed “rarely (<2 hours/day)”, “often (2–4 hours/day)” or “always (≥4 hours/day)”. Using the Rating Perceived Exertion Borg scale (RPE Borg Scale) [52] ranging from 6 (no exertion at all) to 20 (maximal exertion), high perceived physical exertion was defined based on the threshold (RPE ≥12) proposed by the French National Research and Safety Institute for the Prevention of Occupational Accidents and Diseases [53].
3. ***Psychosocial factors*** were assessed using the 26 items of the French version of the Karasek Job Content Questionnaire (JCQ) [54]. High psychological demand, low decision latitude and low social support were defined based on the median values of the national French SUMER study to classify exposed and unexposed workers [55].

### Statistical analysis

Cox regression models with equal follow-up time and robust variance estimates were used to estimate age-adjusted and multivariable-adjusted relative risks (RRs) and their 95% confidence intervals (CIs) [56] in the overall cohort. In addition, we conducted a sensitivity analysis separately for men and women to account possible differences in exposure to work constraints between genders [57]. Multivariable models included only factors significant with a Wald test *p*-value of less than 0.20 in age-adjusted models [58]. Age being recognized as a major risk factor for UEMSD in the literature [33, 59, 60], we decided to force it into multivariable models even if it was not statistically significant in age-adjusted models. Diabetes mellitus and rheumatoid arthritis were excluded from analyses due to a low number of UEMSD cases exposed (less than five UEMSD cases for each gender). Interactions between all occupational exposures have been explored to verify that these risk factors are independent factors in relation to the risk of UEMSD.

### Estimation of the partial population-attributable fractions (PAFs)

Based on the multivariable models, PAFs were estimated separately for each factor included in the multivariable models. The calculation of partial PAF is recommended for multifactorial diseases when some risk factors are unmodifiable or not expected to change after intervention [36]; the calculated PAFs were considered as partial because the set of the risk factors taken into account includes unmodifiable risk factors (in theory) (e.g. age). PAFs express the percentage of UEMSD cases that could have been avoided for each risk factor separately, with the assumption of a causal relationship from the risk factors and if all other risk factors did not change.

For the estimation of PAFs and their CIs, we used the method described by Spiegelman and colleagues [61] with the SAS macro, which is fully-documented and publicly available (https://www.hsph.harvard.edu/donna-spiegelman/software/par/). The CIs were estimated using the multivariable delta method [61, 62] as carried out by Rajaobelina and colleagues [25]. The prevalence of the exposure and adjusted RRs were considered in PAF estimates. The PAF indicated the percentage of UEMSD cases theoretically preventable if all workers were exposed in the lowest risk group. Only PAFs for factors associated with the risk of UEMSD in multivariable models with a *p*-value of ≤10% have been reported in the text.

All statistical analyses were performed using the SAS software, version 9.4 (SAS Institute Inc., Cary, NC, USA).

## Results

### Baseline characteristics

Of the 3170 workers included at baseline, 1228 were excluded from follow-up due to the death, retirement, parental leave, long-term sick leave, unemployment, etc. Among the remainder, 23 refused to participate in the follow-up. In addition, 848 workers did not undergo the second clinical examination because they did not have a mandatory examination scheduled between the time the OP was notified that he/she was in charge for a worker and the end of the follow-up period. A comparison of baseline characteristics of workers with follow-up and workers without follow-up (see Additional file 1, Appendix A) showed a significant difference in age between the workers who were followed up and those lost to follow-up. Workers aged < 35 years and ≥ 45 years were more frequent in those lost to follow-up. Moreover, workers with length of service of < 2 years and temporary workers were more frequent among the workers lost to follow-up.

Of the 1611 workers re-examined during follow-up, 226 were with UEMSD at baseline (prevalent cases). Out of 1385 eligible participants i.e. free of UEMSD at baseline, 95 workers with missing covariates data and 15 workers with an unknown UEMSD diagnosis at follow-up were excluded.

**Fig. 1:**
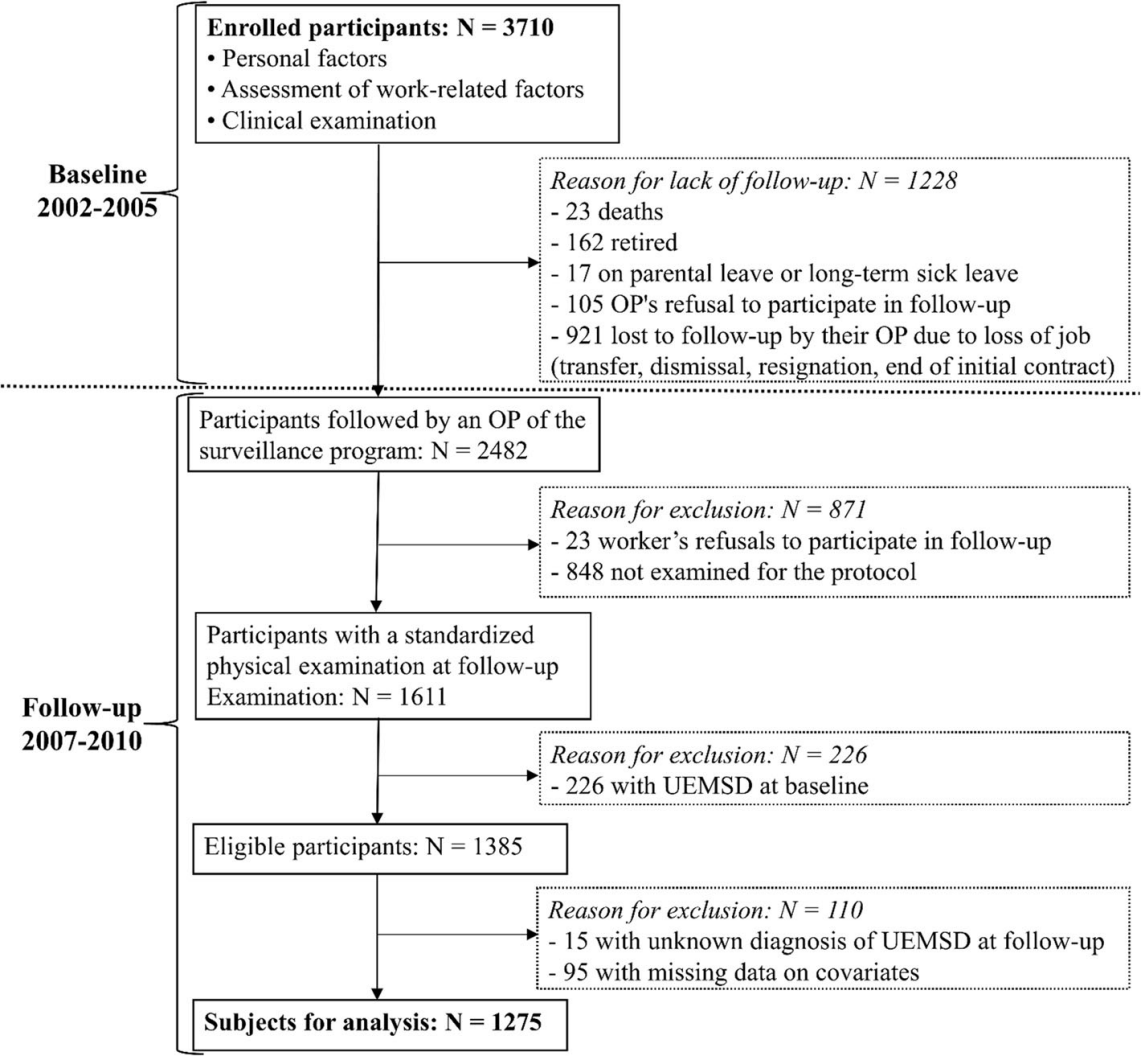
Participants flow diagram

A total of 1275 participants (mean age: 38.2 years, SD = 8.7), 754 (59.1%) men and 521 (40.9%) women, were included in current analyses (Fig. 1). Participants with missing data did not differ with regards to BMI, diabetes, arthritis and seniority in current job, exposures and outcome compared to those with complete data. Participants with missing data were significantly older than those with complete data (*p* = 0.001), were more likely to be low-grade white-collar workers (*p* = 0.008) and were more likely to work in the trade and services sectors (*p* = 0.013) (see Additional file 1, Appendix B).

A description of characteristics and working conditions at baseline according to gender is provided in an additional file (see Additional file 1, Appendix C). The prevalence of being overweight/obese was higher in men than in women (*p* < 0.001). Most men worked as blue-collar workers in the industry sector, while most women were low-grade white-collar workers and worked in the trade and services sectors (p < 0.001). No difference was observed considering the history of diabetes mellitus, rheumatoid arthritis and the seniority in the current job.

### UEMSD at follow up

At least one of the six UEMSD was diagnosed at follow up in 143 workers (76 men and 67 women) out of the 1275 followed (Table 1). The incidence rate of UEMSD observed did not significantly differ between genders (10.1% for men and 12.9% for women; *p* = 0.122). The most common diagnoses at follow-up was RCS (incidence rate 6.4% for the overall population, 5.7% for men and 7.3% for women). LET was more common in men than in women (2.9% vs 1.2% cases, *p* = 0.034) while CTS was more common in women (3.5% vs 0.9% cases; *p* = 0.001). More than one UEMSD was diagnosed at follow-up in 20 workers (incidence rate 1.6%).

**Table 1:** Distribution of the six Upper Extremity Musculoskeletal Disorders (UEMSD) among the study population

	Overall population (***N*** = 1275)		Men (***N*** = 754)		Women (***N*** = 521)		P
	UEMSD	%	UEMSD	%	UEMSD	%	
Rotator cuff syndrome (RCS)	81	6.4	43	5.7	38	7.3	0.255
Lateral epicondylar tendinopathy (LET)	28	2.2	22	2.9	6	1.2	**0.034**
Carpal tunnel syndrome (CTS)	25	2.0	7	0.9	18	3.5	**0.001**
Ulnar tunnel syndrome	12	0.9	7	0.9	5	1.0	0.956
De Quervain tenosynovitis	10	0.8	4	0.5	6	1.2	0.333*
Flexor-extensor peritendinitis or tenosynovitis of the forearm-wrist region	9	0.7	5	0.7	4	0.8	1.000*
At least one of the six UEMSD	143	11.2	76	10.1	67	12.9	0.122
At least two of the six UEMSD	20	1.6	11	1.5	9	1.7	0.704

*P*: Chi-square test for difference between genders; *Fisher’s exact test for difference between genders; *P* < 0.05 are in bold

### Risk factors and UEMSD

Age-adjusted models have shown that personal, biomechanical and, psychosocial risk factors were positively associated with the incident of UEMSD (with a *p*-value less than 20%) (Table 2).

**Table 2:** Age-adjusted models for risk factors of incident UEMSD in the Cosali cohort

	Overall population (N = 1275)				Men (N = 754)				Women (N = 521)			
	UEMSD = 143	RR	95% CI	P	UEMSD = 76	RR	95% CI	P	UEMSD = 67	RR	95% CI	P
**Personal factors**												
Female gender	67	1.25	(0.90–1.73)	**0.188**								
Age: 35–44 years	51	1.38	(0.91–2.11)	**0.129**	28	1.65	(0.91–2.99)	**0.096**	23	1.13	(0.62–2.06)	0.686
Age: ≥45 years	54	1.91	(1.26–2.90)	**0.002**	30	2.46	(1.37–4.42)	**0.003**	24	1.41	(0.78–2.57)	0.249
Overweight/obesity^a^	59	1.20	(0.86–1.68)	0.295	33	0.91	(0.57–1.44)	0.683	26	1.87	(1.14–3.08)	**0.013**
**Biomechanical factors**^b^												
High perceived physical exertion (RPE Borg scale ≥12)^c^	98	2.10	(1.47–2.99)	**< 0.001**	58	2.60	(1.53–4.43)	**< 0.001**	40	1.84	(1.13–3.01)	**0.015**
High repetitiveness of tasks (> 4 h/day)	41	1.54	(1.07–2.22)	**0.020**	16	1.27	(0.73–2.22)	0.398	25	1.79	(1.09–2.95)	**0.021**
Use of vibrating tools (≥2 h/day)	19	1.12	(0.69–1.82)	0.650	17	1.33	(0.77–2.28)	0.307	2	0.86	(0.21–3.50)	0.828
Repeated/sustained posture with arms above shoulder level (≥2 h/day)	27	2.16	(1.42–3.29)	**< 0.001**	14	1.96	(1.09–3.51)	**0.024**	13	2.41	(1.30–4.46)	**0.005**
Repeated/sustained posture with shoulder abduction^d^	57	1.58	(1.13–2.22)	**0.007**	29	1.26	(0.79–2.00)	0.329	28	2.23	(1.37–3.63)	**0.001**
Repeated/sustained elbow movements (flexion/extension) (≥2 h/day)	49	1.36	(0.96–1.93)	**0.079**	29	1.61	(1.01–2.56)	**0.045**	20	1.13	(0.67–1.90)	0.658
Pronation and supination movements (≥2 h/day)	23	1.32	(0.84–2.06)	0.228	18	1.40	(0.82–2.37)	0.216	5	1.47	(0.59–3.65)	0.411
Wrist twisting movements (≥2 h/day)	53	1.39	(0.99–1.96)	**0.057**	24	1.07	(0.66–1.75)	0.780	29	1.92	(1.18–3.11)	**0.009**
Use of the pinch grip (≥4 h/day)	12	1.23	(0.68–2.23)	0.487	4	0.93	(0.34–2.55)	0.885	8	1.47	(0.70–3.09)	0.303
**Psychosocial factors**^e^												
Low social support	64	1.47	(1.06–2.05)	**0.022**	37	1.59	(1.01–2.49)	**0.045**	27	1.39	(0.85–2.27)	**0.184**
Low decision latitude	76	1.14	(0.82–1.59)	0.424	35	1.08	(0.69–1.70)	0.737	41	1.16	(0.71–1.90)	0.554
High psychological demand	71	1.04	(0.75–1.45)	0.802	41	1.35	(0.86–2.11)	**0.198**	30	0.77	(0.48–1.25)	0.295

*P p*-value of Wald test; *P* < 0.20 are in bold; *RR* relative risk; *95% CI* 95% confidence interval, ^a^assessed using the World Health Organization criteria [51], ^b^assessed using exposure criteria from the European consensus criteria for evaluating the work-relatedness of UEMSD [48], ^c^assessed using the RPE Borg scale [52], ^d^Workers were defined as being at risk if they responded “rarely (<2 h/day)”, “often (2–4 h/day)” or “always (≥4 h/day)” [49], ^e^assessed using the French JCQ [55]

In the multivariable models (Table 3), the personal risk factors associated with increased risks of incident UEMSD were female gender (RR = 1.36; (95% CI 1.00–1.85)) and age (RR = 1.55 (1.04–2.29) for the age group 35–44 and, RR = 2.17 (1.47–3.19) for the age group ≥45). The occupational factors positively associated with an increased risk of UEMSD were high perceived physical exertion (RR = 1.80 (1.24–2.62), repeated/sustained posture with arms above shoulder level (RR = 1.59 (1.06–2.37)) and low social support (RR = 1.37 (1.01–1.87)). No interaction was found between occupational exposures.

**Table 3:** Multivariable models for risk factors of incidence of UEMSD in Cosali cohort

	Overall population (N = 1275)				Men (N = 754)				Women (N = 521)			
	UEMSD = 143	RR	95% CI	P	UEMSD = 76	RR	95% CI	P	UEMSD = 67	RR	95% CI	P
**Personal factors**												
Female gender	67	1.36	(1.00–1.85)	**0.049**								
Age: 35–44 years	51	1.55	(1.04–2.29)	**0.030**	28	1.83	(1.04–3.21)	**0.036**	23	1.32	(0.76–2.28)	0.326
Age: ≥45 years	54	2.17	(1.47–3.19)	**< 0.001**	30	2.85	(1.66–4.91)	**< 0.001**	24	1.48	(0.86–2.57)	0.158
Overweight/obesity^a^									26	1.74	(1.10–2.75)	**0.019**
**Biomechanical factors**^b^												
High perceived physical exertion (RPE Borg scale ≥12)^c^	98	1.80	(1.24–2.62)	**0.002**	58	2.27	(1.32–3.92)	**0.003**	40	1.10	(0.60–2.00)	0.759
High repetitiveness of tasks (> 4 h/day)	41	1.22	(0.87–1.70)	0.248					25	1.37	(0.89–2.11)	0.156
Repeated/sustained posture with arms above shoulder level (≥2 h/day)	27	1.59	(1.06–2.37)	**0.024**	14	1.32	(0.74–2.37)	0.348	13	1.60	(0.93–2.74)	0.089
Repeated/sustained posture with shoulder abduction^d^	57	1.16	(0.81–1.64)	0.418					28	1.56	(0.93–2.64)	0.092
Repeated/sustained elbow movements (flexion/extension) (≥2 h/day)	49	1.00	(0.70–1.44)	0.997	29	1.25	(0.79–1.99)	0.334				
Wrist twisting movements (≥2 h/day)	53	0.98	(0.67–1.42)	0.903					29	1.29	(0.76–2.18)	0.341
**Psychosocial factors**^e^												
Low social support	64	1.37	(1.01–1.87)	**0.042**	37	1.41	(0.93–2.15)	0.106	27	1.33	(0.85–2.09)	0.217
High psychological demand					41	1.34	(0.87–2.05)	0.185				

*P p*-value of Wald test; *P* < 0.05 are in bold, *RR* relative risk, *95% CI* 95% confidence interval, ^a^assessed using the World Health Organization criteria [51], ^b^assessed using exposure criteria from the European consensus criteria for evaluating the work-relatedness of UEMSD [48], ^c^assessed using the RPE Borg scale [52], ^d^Workers were defined as being at risk if they responded “rarely (<2 h/day)”, “often (2–4 h/day)” or “always (≥4 h/day)” [49], ^e^assessed using the French JCQ [55]

The sensitivity analysis (Table 3) showed that, in male workers, high perceived physical exertion was associated with an increased risk of UEMSD (RR = 1.80 (1.24–2.62)). The risk of UEMSD associated with low social support was of the borderline of significance (RR = 1.41 (0.93–2.15)). In female workers, being overweight or obese was associated with an increased risk of UEMSD (RR = 1.74 (1.10–2.75)). The association with arms above shoulder level (RR = 1.6 (0.9–2.6)) and shoulder abduction (RR = 1.6 (0.9–2.7)) approached statistical significance but the 95% CI included the value one.

### Partial population attributable fraction (PAF) for UEMSD risk factors

**Fig. 2:**
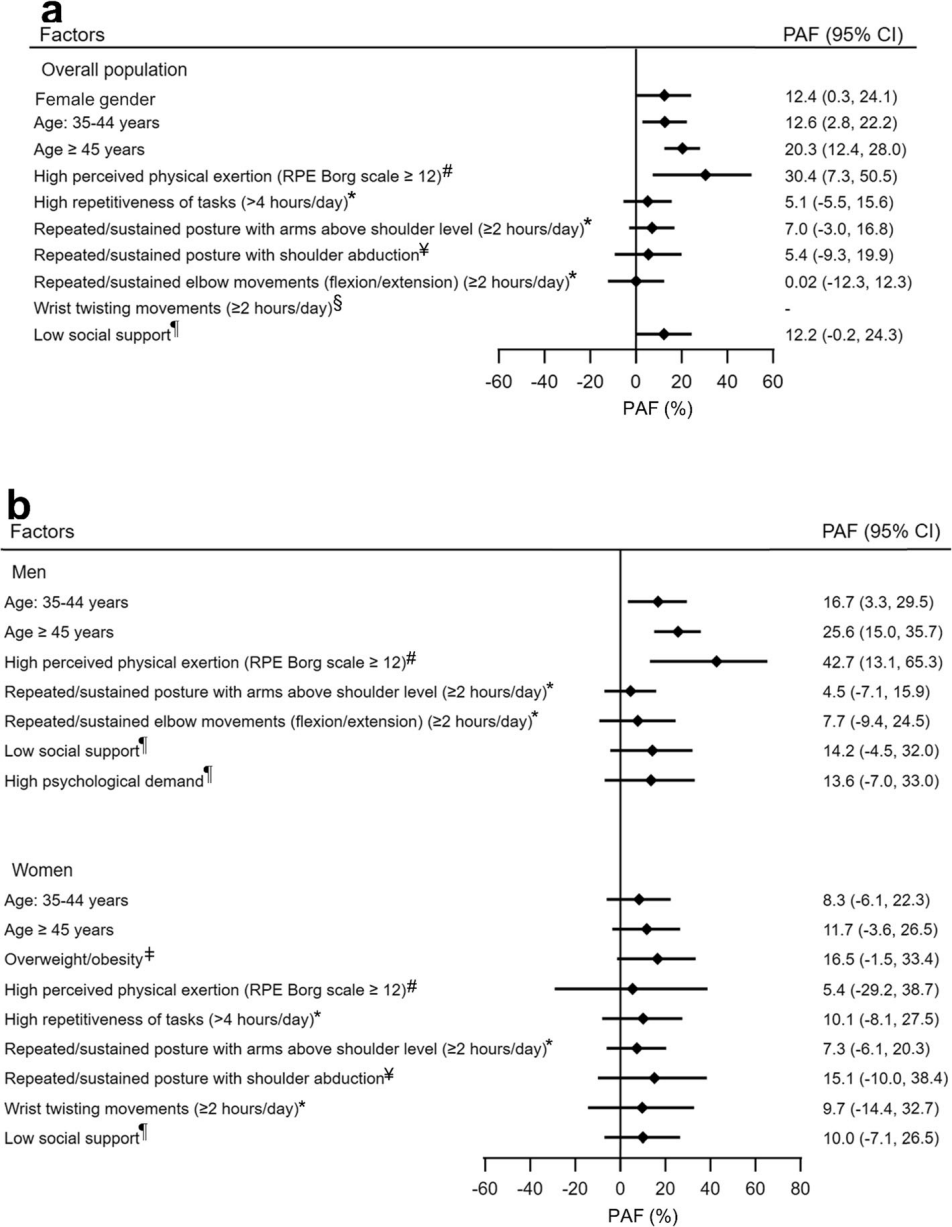
Population-attributable fraction (PAF) for UEMSD risk factors, adjusting for all factors shown, in Cosali cohort. The PAF was calculated using the lowest risk group for each factor as the reference group, with all other factors remaining unchanged. **a** Overall population, **b** Stratify by gender, ^#^assessed using the RPE Borg scale [52], ^*^assessed using exposure criteria from the European consensus criteria for evaluating the work-relatedness of UEMSD [48], ^§^relative risk < 1 and the PAF was not calculated, ^¶^assessed using the French JCQ [55], ^ǂ^assessed using the World Health Organization criteria [51], ^¥^Workers were defined as being at risk if they reported being exposed “rarely (<2 hours/day)”, “often (2–4 hours/day)” or “always (≥4 hours/day)” [49]

Considering the PAF of UEMSD for occupational risk factors, a high perceived physical exertion explained 30% (7 to 51) of cases (Fig. 2a). An estimated of 7% (− 3 to 17) and 12% (− 0.2 to 24) of UEMSD cases were attributable to working with arms above shoulder level (≥2 h/day) and low social support, respectively. Concerning personal risk factors PAFs were of 12% (95% CI: 0.3 to 24) for female gender, 13% (3 to 22) for the age group 35–44, and 20% (12 to 28) for the age group ≥45.

Sensitivity analysis (Fig. 2b) in men showed that a high perceived physical exertion was the leading risk factor with a PAF of 43% (95% CI: 13 to 65), followed by the age group 35–44 (PAF: 26% (15 to 36)), the age group 35–44 (PAF: 17% (3 to 30)) and, low social support (PAF: 14% (− 5 to 32)). In women, the PAF was of 17% (− 2 to 33) for being overweight or obese, followed by working posture with shoulder abduction (15% (− 10 to 38)) and, working posture with arms above shoulder level (7% (− 6 to 20)).

## Discussion

For multifactorial diseases, such as UEMSD, the PAFs allows an estimation of the contribution of the work-related and non-work-related risk factors to the burden of disease in the working population. In the multivariable models, our results showed that the main risk factors of UEMSD were, in decreasing order, high perceived physical exertion (PAF: 30%), the age group ≥45 (PAF: 20%), age group 35–44 (PAF: 13%), female gender (PAF: 12%), low social support (PAF: 12%) and, working with arms above shoulder level (PAF: 7%).

### Literature comparison

There are extensive literature demonstrating the links between personal factors and work-related risk factors and UEMSD [2, 7, 8, 10, 11, 14–18, 50, 63–72]. Most studies of provided RRs or odds ratios associated with these personal or work-related risk factors. Only few studies assessed the proportion of UEMSD cases attributable to these risk factors and their contribution to the burden of UEMSD in the working population [28, 30, 73]. Most of them [2, 29, 30, 74] have quantified the impact of work-related exposures on the occurrence of UEMSD only in exposed population. One study [28] estimated PAFs for CTS ranging from 19 to 50% according to occupational categories and from 5 to 17% according to industrial sectors.

In our study, age ≥ 45 years contributed importantly to the incidence of UEMSD: 20% of cases of UEMSD that could be attributed to this age group. However, this major personal factor is unmodifiable. In this case, age can be considered as a marker of the degenerative process of the periarticular soft tissues, but also as a marker of the cumulative exposure of work-related risk factors [75]. Female gender was associated with 12% of UEMSD cases occurring in the working population. This PAF was lower than the one (34%) associated with CTS in a cohort study conducted in Italy (OCTOPUS study) [33].

Considering work-related exposures, our results showed that the risk of UEMSD was associated with high physical exertion, working with arms above shoulder level and, low social support. These findings are consistent with a large body of epidemiologic studies that evaluated the relation between UEMSD and occupational exposures [7, 8, 11, 14, 16–18, 66, 67, 71, 76, 77].

Regarding PAFs, we found high PAFs for both biomechanical and psychosocial factors, after adjustment for personal risk factors. Concerning biomechanical factors, findings suggested that nearly 30% of UEMSD could potentially be avoided by lowering the physical exertion on the RPE Borg scale below 12 (RPE Borg scale range = 6 to 20). The United States National Research Council and Institute of Medicine [2] report on MSD estimated an attributable fraction (AF) for work-related upper extremity disorders risk in exposed population at the workplace. AF estimates were 78% for high forces, between 28 and 52% for low social support, and between 33 and 58% for high psychological demand, based on how specifically the exposure and the outcome were defined. A Canadian study [30] estimated AF in exposed people by comparing the incidence of CTS surgery among different working groups, using non-manual workers as the reference population. Among manual workers in Montreal, 55% of surgical CTS in women and 76% in men were attributable to work. A Swedish study based on the review of epidemiologic studies [74] concluded that at least 50%, and as much as 90%, of all of the CTS cases in working populations exposed to physical work load factors such as repetitive and forceful gripping appeared to be attributable to physical work load. A French study [29] has shown that a proportion of CTS ranging between 36 and 93% could be attributed to industry sectors and occupational categories. The findings of these studies support the results observed in the working population characterized by a high contribution of work-related factors, especially in male workers. The current study found a PAF of 7% that could be attributed to working with arms above shoulder level. A prospective cohort study of Harkness et al. [78] found an association between new of onset shoulder pain and working with hands above shoulder. A recent systematic review and meta-analysis [16] have revealed moderate evidence for associations between shoulder disorders and arm-hand elevation.

For psychosocial factors in this study, we demonstrated that 12% of UEMSD cases could be attributed to low social support, although the PAF did not reach the statistical level of significance. However, epidemiologic investigations have demonstrated the relationship between some types of UEMSD and work-related psychosocial factors. A systematic literature review of van Rijn et al. [17] showed that psychosocial factors including low social support at work were associated with an increased occurrence of LET. Moreover, a pooled study cohort [68] has reported that workers with high social support in the workplace had half the risk of CTS incidence compared with those with low social support.

Sensitivity analyses were stratified by gender to account possible differences in personal risk factors and exposure to occupational hazards exposure between men and women [57]. In men, factors affected the risk of UEMSD were high perceived physical exertion, low social support, and age. In women, these factors were working with shoulder abduction, working with arms above shoulder level, and overweight/obese. The OCTOPUS study found a PAF of 30% for being overweight/obese associated with CTS risk [33]. Several patho- or biomechanical mechanisms might be involved in relationship between overweight/obesity and the risk of UEMSD in women. Obesity may increase the risk of CTS [68, 79], a disorder more frequent in the women in our study, due to the accumulation of fat tissue within the carpal tunnel; this has been hypothesized to increase intra-carpal tunnel pressure [80, 81]. Secondly, obesity may increase the risk of rotator cuff tendinopathy [82, 83] and LET [84] due to failure of tendon repair in obese workers. Another explanation is that severe obesity may modify the worker’s anthropometric characteristics leading to (i) increased shoulder abduction at rest and in activity and (ii) increased moment of forces applied on the shoulder joint and rotator cuff tendons due to increased weight of the upper limb [85]. Such mechanisms may be particularly important in workers who are exposed to high physically demanding jobs [86]. Our study found a noticeable PAF value (15%) for working with shoulder abduction in women, even if this result was not statistically significant possibly due lack of statistical power (only 38 cases of RCS). This PAF estimate is consistent with recent meta-analyses showing increased risks of rotator cuff tendinopathy with shoulder abduction [16, 87].

### Strengths and limitations

There were some potential limitations of our study that could have affected the results. Of the 3710 workers initially included, about 57% (young workers, those in short-time working or with a short period of service) did not undergo the follow-up clinical examination. According a longitudinal study of MSD [88], differences in occupational conditions between participants and those lost to follow-up did not significantly influence estimates of risk ratios. Diabetes mellitus and rheumatoid arthritis which are associated with UEMSD in the literature were not studied due to a low number of UEMSD cases exposed (less than five UEMSD cases in each gender) (see Additional file 1, Appendix C).

The thresholds used to define exposure levels may influence PAF estimates [89]. However, these cut-points were chosen based on the literature and public health recommendations. A second limitation is that the assessment of exposures was based on self-reported exposure, whereas assessing UEMSD cases was based on clinical examination. The non-differential misclassification of exposures may have occurred due to workers’ inability to precisely recall or describe their current work exposures. Lack of measurement precision may also have occurred when quantifying exposures due to the 1 to 4 point ordinal scale used in exposure questions, except for physical exertion which was assessed using the RPE Borg Scale [52]. To the extent that the risk of UEMSD is increased by cumulative or chronic physical exposures, our analyses may have underestimated the true contribution of work exposures to the incidence of UEMSD in our study population. This may be especially true for RCS, as studies of occupational risk factors for shoulder pain have consistently identified duration of employment as a risk factor [90]. Furthermore, it’s important to note that the PAF associated with single risk factors cannot be added to obtain a combined PAF associated with a combination of risk factors and that a combined PAF cannot be subtracted from 100% to determine the “unexplained” proportion of cases [26]. Further studies on a larger sample could appropriately assess the PAF associated with co-exposures. Despite the importance of PAF estimates, which are useful to rank risk factors, we should note that public health interventions are not possible for all factors (e.g. age) and a total elimination of risk factors in the population level is practically impossible. Finally, we should note that, the estimation of PAFs was assumed to have a causal relationship between exposure and UEMSD and should therefore be interpreted with caution.

The study has also several strengths. A major strength is that the study included a representative sample of the working population at baseline. Secondly, the definition of incidence cases was based on a standardized clinical examination performed by a trained occupational physician. Due to the prospective design of the study, exposure information gathered prior to UEMSD diagnosis resulted in low risk of recall bias. Another strength is the formula used to estimate the PAF from multivariable regression models, allowing a non-biased estimation of adjusted PAF [26]. This regression-based PAF estimation method allows to control confounding and interaction, and can be used for the main epidemiologic designs [91].

## Conclusions

Our study suggests that an important fraction of UEMSD can be attributed to occupational exposures such as physical exertion and low social support, after the contributions of personal and other work-related factors are considered. Potentially modifiable personal factors, such as being overweight or obesity, contribute to the population burden of UEMSD in women. Despite the lack statistical significance of the PAF associated with factors such as working with shoulder abduction in women, interventions should still consider these factors recognized in literature as being associated with UEMSD. In terms of public health, the findings of the present study are in agreement with the ergonomic literature postulating that a high proportion of UEMSD are preventable through modifying workplace risk factors [92, 93]. Interventions should still consider recognizable risk factors that have been found to be associated with UEMSD in the literature. Such information is useful to help public health practitioners and policy makers implement programs of prevention of UEMSD in the working population.

## Supplementary information

**Additional file 1 : Appendix A.** Comparison of baseline characteristics of workers with follow-up and workers without follow-up. **Appendix B.** Comparison of baseline characteristics, outcome and working conditions between respondents with complete and missing data. **Appendix C.** Characteristics and working conditions of the study population at baseline according to gender.

## Data Availability

The datasets used and/or analyzed during the current study are available from the corresponding author on reasonable request.

## Abbreviations

UEMSD: Upper extremity musculoskeletal disorders
RCS: Rotator cuff syndrome
CTS: Carpal tunnel syndrome
LET: Lateral epicondylar tendinopathy
OP: Occupational physician
BMI: Body mass index
RPE: Rating perceived exertion
RR: Relative risk
95% CI: 95% confidence interval
PAF: Population-attributable fraction
WHO: World Health Organization
