# Supplementary material for: Proportion of upper extremity musculoskeletal disorders attributable to personal and occupational factors: results from the French Pays de la Loire study
Source: BMC Public Health. 2020 Apr 6;20:456. doi: 10.1186/s12889-020-08548-1 (PMC7137334; doi:10.1186/s12889-020-08548-1)
Supplement: Supplementary file 1 — Additional file 1 : Appendix A. Comparison of baseline characteristics of workers with follow-up and workers without follow-up. Appendix B. Comparison of baseline characteristics, outcome and working conditions between respondents with complete and missing data. Appendix C. Characteristics and working conditions of the study population at baseline according to gender. [file 12889_2020_8548_MOESM1_ESM.pdf]

Supplementary Tables

Additional file 1, Appendix A: Comparison of baseline characteristics of workers with follow-up and workers without follow-up

	Overall population (N=3710)				
	With follow-up (N=1611)		Without follow-up (N=2099)		p
	n	%	n	%	
Gender					0.243
Men	921	57.2	1240	59.1	
Women	690	42.8	859	40.9	
Age (years)					<0.001
<35	533	33.1	892	42.5	
35-44	568	35.3	492	23.5	
≥45	510	31.7	714	34.0	
Overweight/obesity	579	36.5	799	38.6	0.199
Diabetes mellitus	17	1.1	44	2.1	0.013
Rheumatoid arthritis	29	1.8	49	2.3	0.259
Seniority in current job (years)					<0.001
<1	130	8.1	325	15.7	
1-2	235	14.7	356	17.2	
3-10	573	35.9	665	32.0	
>10	660	41.3	729	35.1	
Occupational class					0.055
Craftsmen, salesmen and managers	6	0.4	10	0.5	
Professionals	114	7.1	174	8.3	
Technicians, associate professionals	378	23.5	451	21.5	
Low-grade white-collar workers	455	28.3	531	25.3	
Blue-collar workers	656	40.8	930	44.4	
Temporary employment	93	5.8	333	15.9	<0.001
Economic sector					<0.001
Agriculture	13	0.8	58	2.8	
Industry	599	37.2	623	29.7	
Construction	80	5.0	134	6.4	
Trade and services	918	57.0	1282	61.1	
High perceived physical exertion (RPE Borg scale ≥ 12)	877	54.7	1201	57.6	0.082
High repetitiveness of tasks (>4 hours/day)	396	24.8	562	27.0	0.138
Use of vibrating tools (≥2 hours/day)	204	12.7	265	12.7	0.982
Repeated/sustained posture with arms above shoulder level (≥2 hours/day)	190	11.8	297	14.2	0.035
Repeated/sustained posture with shoulder abduction	525	32.7	731	35.0	0.139
Repeated/sustained elbow movements (flexion/extension) (≥2 hours/day)	495	30.9	719	34.5	0.020
Pronation and supination movements (≥2 hours/day)	217	13.5	317	15.2	0.161

Wrist twisting movements (≥2 hours/day)	523	32.8	713	34.4	0.311
Use of the pinch grip (≥4 hours/day)	127	7.9	170	8.1	0.804
Low social support	577	36.8	831	40.9	0.011
Low decision latitude	768	48.3	978	47.3	0.566
High psychosocial demand	785	49.1	1030	49.8	0.668
**P**: p-value of independent Khi-2 test					

Additional file 1, Appendix B: Comparison of baseline characteristics, outcome and working conditions between respondents with complete and missing data

	With complete data (n = 1275)		With missing data (n = 110)		P
	n	%	n	%	
**Baseline characteristics**					
Gender					0.003
Men	754	59.1	49	44.5	
Women	521	40.9	61	55.5	
Age (years)					0.001
<35	470	36.9	25	22.7	
35-44	456	35.8	39	35.5	
≥45	349	27.4	46	41.8	
Overweight/obesity	447	35.3	33	31.7	0.469
Diabetes mellitus	13	1	2	1.8	0.335*
Rheumatoid arthritis	20	1.6	2	1.8	0.691*
Seniority in current job (years)					0.091
<1	114	9	7	6.5	
1-2	198	15.7	15	14	
3-10	470	37.2	31	29	
>10	483	38.2	54	50.5	
Occupational class					0.008
Craftsmen, salesmen and managers	3	0.2	0	0	
Professionals	96	7.5	5	4.6	
Technicians, associate professionals	310	24.3	24	22.2	
Low-grade white-collar workers	348	27.3	47	43.5	
Blue-collar workers	518	40.6	32	29.6	
Temporary employment	77	6.4	9	8.3	0.349
Economic sector					0.013
Agriculture	12	0.9	1	0.9	
Industry	483	37.9	24	22	
Construction	66	5.2	6	5.5	
Trade and services	714	56	78	71.6	
**Working conditions at baseline**					
**Biomechanical factors**					
At least one of the six UEMSD[#]	143	11.2	9	9.5	0.602
High perceived physical exertion (RPE Borg scale ≥ 12)	668	52.4	58	56.3	0.444
High repetitiveness of tasks (>4 hours/day)	276	21.6	27	28.1	0.14
Use of vibrating tools (≥2 hours/day)	160	12.5	8	7.6	0.138
Repeated/sustained posture with arms above shoulder level (≥2 hours/day)	132	10.4	12	11.5	0.704
Repeated/sustained posture with shoulder abduction	386	30.3	36	33.6	0.467
Repeated/sustained elbow movements (flexion/extension) (≥2 hours/day)	364	28.5	28	26.7	0.681
Pronation and supination movements (≥2 hours/day)	163	12.8	13	12.6	0.962

Wrist twisting movements (≥2 hours/day)	397	31.1	29	29.9	0.799
Use of the pinch grip (≥4 hours/day)	89	7	5	4.8	0.398
Low social support	456	35.8	28	34.6	0.827
Low decision latitude	638	50	51	54.8	0.372
High psychosocial demand	625	49	42	42.4	0.206
**P**: p-value of independent Khi-2 test; *Fisher's exact test; #UEMSD = Upper Extremity Musculoskeletal Disorders					

Additional file 1, Appendix C: Characteristics and working conditions of the study population at baseline according to gender

	Men (N = 754)		Women (N = 521)		p
	n	%	n	%	
**Baseline characteristics**					
Age (years)					0.295
<35	288	38.2	182	34.9	
35-44	271	35.9	185	35.5	
≥45	195	25.9	154	29.6	
Overweight/obesity	318	42.6	129	24.8	<0.001
Diabetes mellitus	8	1.1	5	1	0.861
Rheumatoid arthritis	10	1.3	10	1.9	0.401
Seniority in current job (years)					0.899
<1	67	9	47	9.1	
1-2	112	15	86	16.6	
3-10	279	37.4	191	36.8	
>10	288	38.6	195	37.6	
Occupational class					<0.001
Craftsmen, salesmen and managers	3	0.4	0	0	
Professionals	67	8.9	29	5.6	
Technicians, associate professionals	204	27.1	106	20.3	
Low-grade white-collar workers	65	8.6	283	54.3	
Blue-collar workers	415	55	103	19.8	
Temporary employment	49	6.5	28	5.4	0.407
Economic sector					<0.001
Agriculture	8	1.1	4	0.8	
Industry	354	46.9	129	24.8	
Construction	59	7.8	7	1.3	
Trade and services	333	44.2	381	73.1	
**Working conditions at baseline**					
**Biomechanical factors**					
High perceived physical exertion (RPE Borg scale ≥ 12)	432	57.3	236	45.3	<0.001
High repetitiveness of tasks (>4 hours/day)	145	19.2	131	25.1	0.012
Use of vibrating tools (≥2 hours/day)	142	18.8	18	3.5	<0.001
Repeated/sustained posture with arms above shoulder level (≥2 hours/day)	81	10.7	51	9.8	0.583
Repeated/sustained posture with shoulder abduction	258	34.2	128	24.6	0.0002
Repeated/sustained elbow movements (flexion/extension) (≥2 hours/day)	219	29	145	27.8	0.637
Pronation and supination movements (≥2 hours/day)	135	17.9	28	5.4	<0.001
Wrist twisting movements (≥2 hours/day)	247	32.8	150	28.8	0.133
Use of the pinch grip (≥4 hours/day)	46	6.1	43	8.3	0.138
**Psychosocial factors**					
Low social support	286	37.9	170	32.6	0.052

Low decision latitude	338	44.8	300	57.6	<0.001
High psychosocial demand	358	47.5	267	51.2	0.186
**P**: p-value of Chi-square test					
